# Examining the effects of cigarette smoke on mouse lens through a multi OMIC approach

**DOI:** 10.1038/s41598-021-95013-7

**Published:** 2021-09-22

**Authors:** Shahid Y. Khan, Muhammad Ali, Yura Jang, Taekyung Ryu, Andrew J. Schwab, Brian O. Ingram, Peter H. Cable, Chan Hyun Na, James T. Handa, S. Amer Riazuddin

**Affiliations:** 1 The Wilmer Eye Institute, Johns Hopkins University School of Medicine, Baltimore, MD 21287 USA; 2 Department of Neurology, Institute for Cell Engineering, Johns Hopkins University School of Medicine, Baltimore, MD 21205 USA; 3 Metabolon Inc, Morrisville, NC 27560 USA; 4 Department of Environmental Sciences and Engineering, University of North Carolina, Chapel Hill, NC 27599 USA

**Keywords:** Molecular biology, Diseases

## Abstract

Here, we report a multi OMIC (transcriptome, proteome, and metabolome) approach to investigate molecular changes in lens fiber cells (FC) of mice exposed to cigarette smoke (CS). Pregnant mice were placed in a whole-body smoke chamber and a few days later pups were born, which were exposed to CS for 5 hours/day, 5 days/week for a total of 3½ months. We examined the mice exposed to CS for CS-related cataractogenesis after completion of the CS exposure but no cataracts were observed. Lenses of CS-exposed and age-matched, untreated control mice were extracted and lens FC were subjected to multi OMIC profiling. We identified 348 genes, 130 proteins, and 14 metabolites exhibiting significant (p < 0.05) differential levels in lens FC of mice exposed to CS, corresponding to 3.6%, 4.3%, and 5.0% of the total genes, protein, and metabolites, respectively identified in this study. Our multi OMIC approach confirmed that only a small fraction of the transcriptome, the proteome, and the metabolome was perturbed in the lens FC of mice exposed to CS, which suggests that exposure of CS had a minimal effect on the mouse lens. It is worth noting that while our results confirm that CS exposure does not have a substantial impact on the molecular landscape of the mouse lens FC, we cannot rule out that CS exposure for longer durations and/or in combination with other morbidities or environmental factors would have a more robust effect and/or result in cataractogenesis.

## Introduction

Cataract is a clouding or opacity of an otherwise transparent lens of the eye. Cataracts can be classified into the following two types: congenital cataracts caused by mutations in genes essential for development of the lens and maintenance of its transparency^1–3^, and age-related cataracts, a multifactorial disorder involving genetic susceptibility loci and environmental factors i.e., cigarette smoke (CS), and ultraviolet (UV) exposure, etc.^4–6^. Age-related cataract represents a significant burden of blindness worldwide that will grow as the age expectancy and population increases globally^7^.

CS has been identified as an important risk factor for development of cataracts^8,9^; however, the molecular mechanism of this association remains elusive. CS is a complex mixture of metal ions and different compounds responsible for the generation of reactive oxygen species (ROS)^10^. Trace and heavy metals have been reported in rat lens exposed to tobacco smoke^11^. In a recent study *p*-benzoquinone induced changes are reported as a causative factor of CS-related cataractogenesis in guinea pig lens^12^.

The ocular lens consists of two, morphologically distinct, cell subpopulations: a monolayer of epithelial cells on the anterior side of the lens, and terminally differentiated fibers cells (FC) that account for the majority of lens volume^13^. Lens FC are generated throughout life by differentiation of lens epithelial cells; however, since lens FC are anuclear and organelle-free, they do not have the ability to repair, and therefore, damage to lens FC results in the opacification of an otherwise transparent lens^13^.

Here, we adopted a multi OMIC (transcriptome, proteome, and metabolome) approach to investigate molecular changes in lens FC of mice exposed to CS. We identified 348 genes, 130 proteins and 14 metabolites exhibiting significant (p < 0.05) differential levels including diminished levels of the branched-chain amino acid (BCAA)-related metabolites in lens FC of mice exposed to CS. To the best of our knowledge, this is the first report of a multi OMIC approach to investigate changes at a molecular level in lens FC of mice exposed to CS.

## Results and discussion

**Figure 1:**
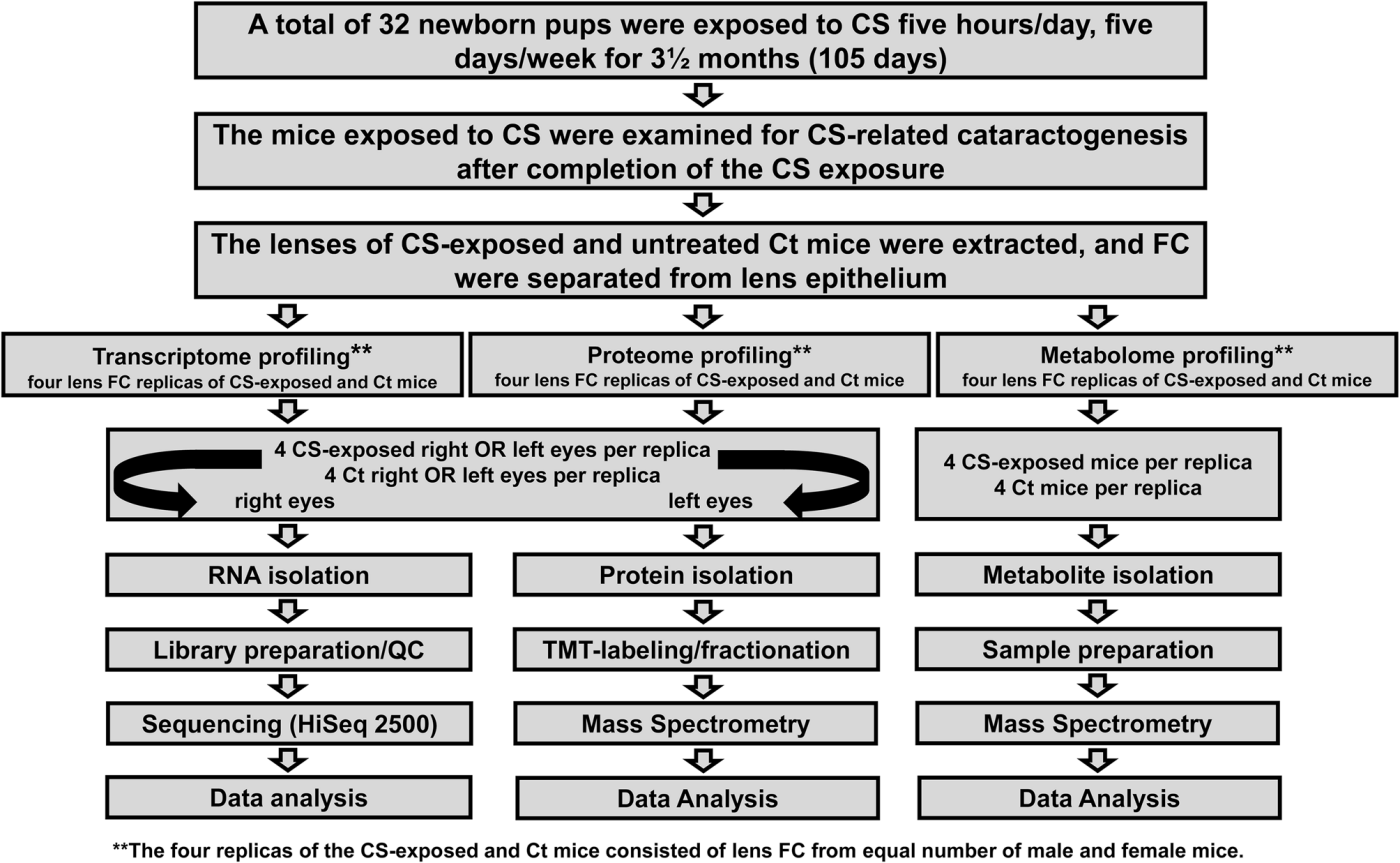
Illustration of the work flow adopted to investigate the effects of cigarette smoke (CS) on mouse lens fiber cells (FC) through a multi OMIC (transcriptome, proteome, and metabolome) approach. A total of 32 newborn mice were exposed to CS in a smoke chamber for 5 hours/day, 5 days/week for a total of 3½ months (105 days). The mice exposed to CS exposure were examined for CS-related cataractogenesis. The CS-exposed and age-matched, untreated control (Ct) mice were euthanized, lenses were extracted, lens FC were separated from the epithelium and used for next-generation RNA sequencing-based transcriptome, mass spectrometry-based proteome, and mass spectrometry-based metabolome profiling. The replicas of FC samples from both CS-exposed and untreated Ct mice consisted of lens from equal number of male and female mice.

**Figure 2:**
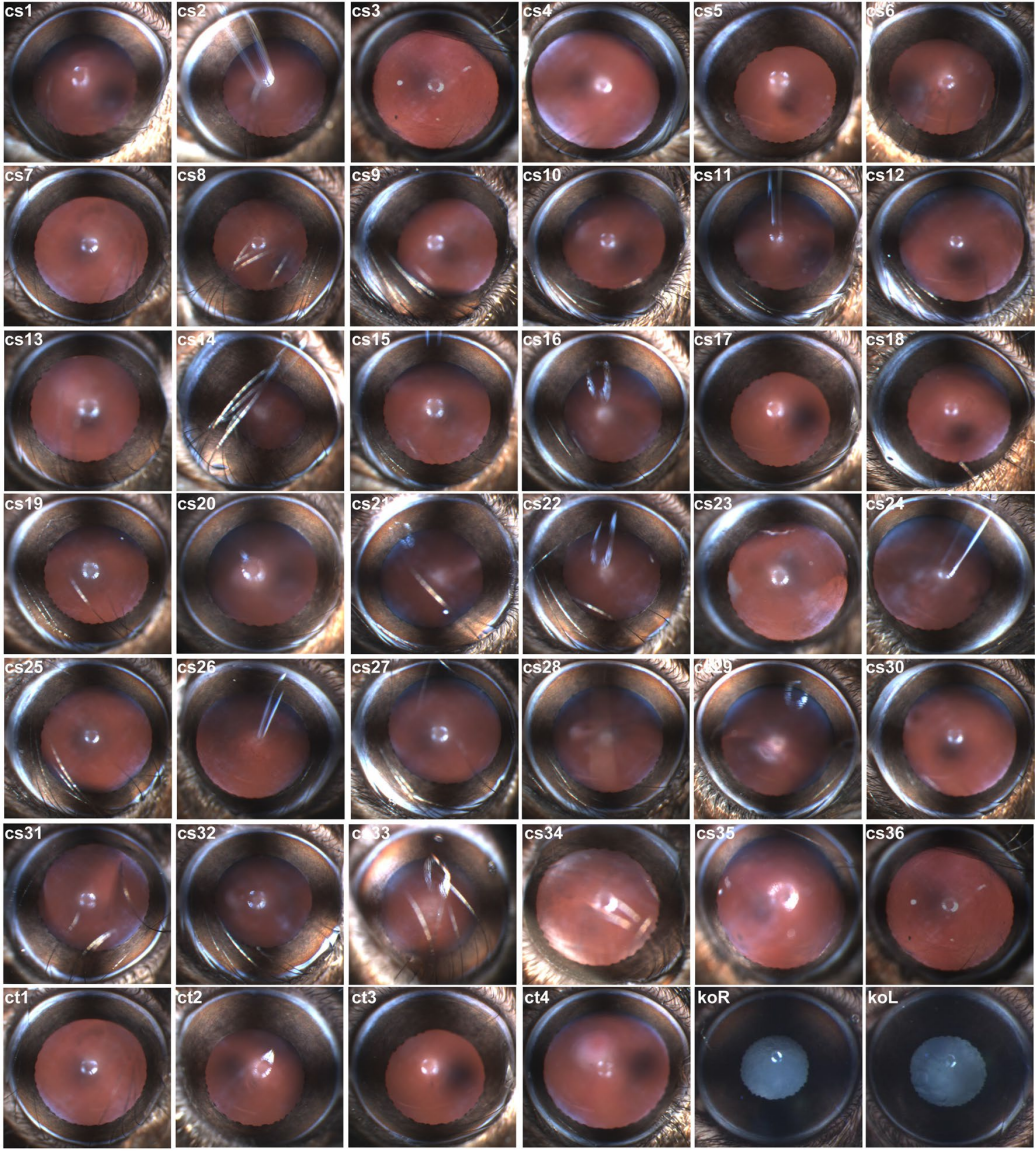
Evaluation of lens phenotype in cigarette smoke (CS)-exposed and age-matched, untreated control (Ct) mice. The 32 mice exposed to CS were examined for CS-related cataractogenesis after completion of CS exposure and age-matched untreated Ct mice but no cataracts were observed. The images of the lens of mice exposed to CS (CS1-32) were similar to images of the lens of age-matched, untreated Ct mice (Ct1-4)﻿. The four adult female mice exposed to CS were examined, 9-months post-exposure for development of cataracts but no cataracts were observed in these mice (CS33-36). The koR and koL illustrate the cataractous lens (right and left eyes, respectively) from a cataract-causing gene knockout mouse.

Here, we evaluate changes at a molecular level in lens FC of mice exposed to CS through a multi OMIC approach (Fig. 1). Pregnant mice (gestation days 18–20) were placed in a whole-body smoke chamber and 2–3 days later pups were born. A total of 32 pups (16 males and 16 females) along with four adult female mice were exposed to CS for 5 hours/day, 5 days/week for a total of 3½ months. In parallel, age-matched, four adult female mice and 32 (16 males and 16 females) pups were housed in the animal facility, hereafter referred to as untreated control (Ct) mice. We examined the mice exposed to CS for CS-related cataractogenesis after completion of the CS exposure but no cataracts were observed. The images of the lens of mice exposed to CS (Fig. 2, CS1-32) were similar to images of the lens of untreated Ct mice (Fig. 2, CS1-4). To investigate if mice exposed to CS develop cataracts over time, we examined the lens of the four adult female mice exposed to CS, 9-months post-exposure but no cataracts were observed in these mice (Fig. 2, CS33–36).

We performed next-generation RNA sequencing (RNA-Seq)-based transcriptome, mass spectrometry-based proteome, and mass spectrometry-based metabolome profiling of lens FC exposed to CS. The lenses were extracted from CS-exposed and untreated Ct mice and FC were separated from lens epithelium under a microscope. The extracted lens FC were maintained in distinct pools from either four right or four left eyes to serve as biological replicates. As illustrated in Fig. 1, the right eyes were used for transcriptome while the corresponding left eyes were used for proteome profiling. As 25 mg of lens FC mass is required for the metabolome analysis, each biological replicate from either CS-exposed or untreated Ct mice consisted of lens FC from both eyes of four mice.

**Figure 3:**
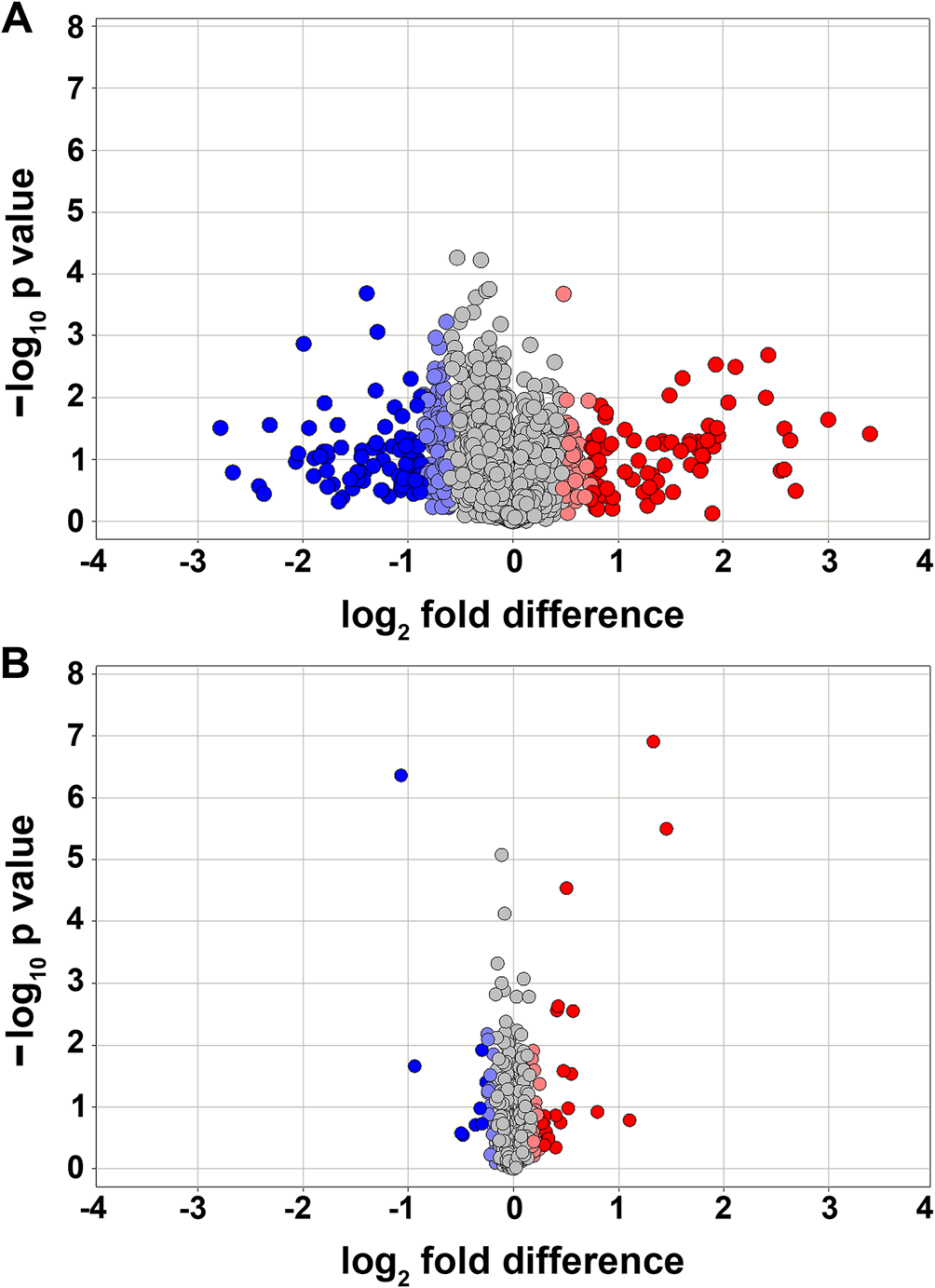
Illustration of genes and proteins perturbed in mouse lens fiber cells (FC) exposed to cigarette smoke (CS). (**A)** Volcano plot of differentially expressed (DE) genes in lens FC of mice exposed to CS. The transcriptome profiling identified 332 DE genes (> ± 2 SD), including 201 down-regulated and 131 up-regulated in FC of mice exposed to CS. (**B)** Volcano plot illustrating differential protein levels in lens FC of mice exposed to CS. The proteome profiling identified 101 proteins (> ± 2 SD) including 59 exhibiting elevated levels and 42 displaying diminished levels in FC of mice exposed to CS. The fold changes are represented in the log2 scale depicted on the x-axis, whereas the − log10 p-value is depicted on the y-axis (the use of -log values mean that genes/proteins with greater statistical significance are higher in the plot). The genes and proteins that are significantly elevated are highlighted in red and light red, and those with significantly diminished levels are highlighted in blue and light blue.

The next-generation sequencing resulted in 424.07 × and 397.36 × sequence coverage for lens FC of CS-exposed and untreated Ct mice, respectively. The mapped reads were assembled into transcripts, and gene expression was measured and normalized using the fragment per kilobase per million mapped reads (FPKM) method, which identified the expression (≥ 1.0 FPKM) of 9531 and 9590 genes in lens FC of CS-exposed and untreated Ct mice, respectively (Supplementary Data 1). The analysis identified 348 differentially expressed (DE) genes (p < 0.05), including 186 down- and 162 up-regulated genes in lens FC of mice exposed to CS (Supplementary Data 1). Moreover, an additional analysis was performed to determine the differential gene expression in lens FC of mice exposed to CS. The standard deviation (SD) was calculated for each transcript expression fold change (log2) as a deviation from its mean of 0 or no change. The analysis revealed 332 DE genes ﻿(> ± 2 SD), including 201 down- and 131 up-regulated genes in lens FC of mice exposed to CS (Fig. 3A). *Col4a4*, *Col4a5*, and *Col6a3* were among the down-regulated genes in lens FC of mice exposed to CS (Supplementary Data 1). Mutations in *Col4a4* and *Col4a5* result in Alport syndrome along with developmental cataracts. Additionally, we identified the differential expression of *Prmt3*, *Rarb*, *Caprin2*, *Gdi2*, *Oat*, *Herc1*, and *Agl* in lens FC of mice exposed to CS (Supplementary Data 1). It is worth noting that loss of *Prmt3*, *Rarb*, *Caprin2*, *Gdi2*, *Oat*, *Herc1*, and *Agl* have been associated with abnormal lens FC morphology and cataracts in mice (http://www.informatics.jax.org/).

In parallel, mass spectrometry generated a total of 84,063 peptide spectrum matches (PSMs), yielding 23,882 total peptides, corresponding to 2323 proteins. We had previously reported 5404 proteins in the mouse lens proteome^14^, and since the number of proteins identified here was significantly less (i.e., 2323 vs. 5404), we performed a second 8-plex TMT experiment to rule out the possibility of a technical error. We detected 2352 proteins in the second TMT experiment, a nearly similar protein count identified in the first TMT experiment. Perhaps, the age of the mice i.e., embryonic, and early postnatal lens compared to 3 months old mice lens and/or the use of the whole lens (lens epithelium and FC used in previously reported study) compared to lens FC used in the current study may have contributed to the difference in protein counts.

The combined analysis of both proteome datasets identified a total of 130 proteins with differential levels (p < 0.05), including 42 exhibiting elevated and 88 diminished levels in lens FC of mice exposed to CS (Supplementary Data 2). To further validate the DE protein, we reanalyzed the proteome datasets by calculating the SD for each protein fold change (log2) as a deviation from its mean of 0 or no change. The analysis revealed 101 DE proteins (> ± 2SD), including 59 exhibiting elevated and 42 diminished levels in lens FC of mice exposed to CS (Fig. 3B). We identified the elevated levels of CTNND2, a delta catenin protein in lens FC of mice exposed to CS (Supplementary Data 2) that has recently been associated with age-related cortical cataracts^15^. Additionally, we identified a decreased levels of CHORDC1, CRYGB, GCLC, HSD17B4, PANK4, PCBD1, OARD1, and HIP1R in lens FC of mice exposed to CS (Supplementary Data 2). Loss of CHORDC1, CRYGB, GCLC, HSD17B4, PANK4, PCBD1, OARD1, and HIP1R has been associated with abnormal lens morphology and cataracts in mice (http://www.informatics.jax.org/). The proteome also revealed diminished levels of GCLC, PCBD1, CRYGB, and PANK4 in lens FC of mice exposed to CS (Supplementary Data 2). Fan and colleagues recently identified nuclear cataracts in lens-specific *Gclc* knockout mice^16^. Bayle and colleagues reported hyperphenylalaninemia and cataracts in *Pcbd1* knockout mice^17^. Liu et al. reported a point mutation in *Crygb* responsible for dominant nuclear cataracts in mice^18^. Sun and colleagues reported a mutation in *PANK4* liable for congenital posterior cataracts and importantly the *Pank4* null mice develop cataracts^19^.

**Figure 4:**
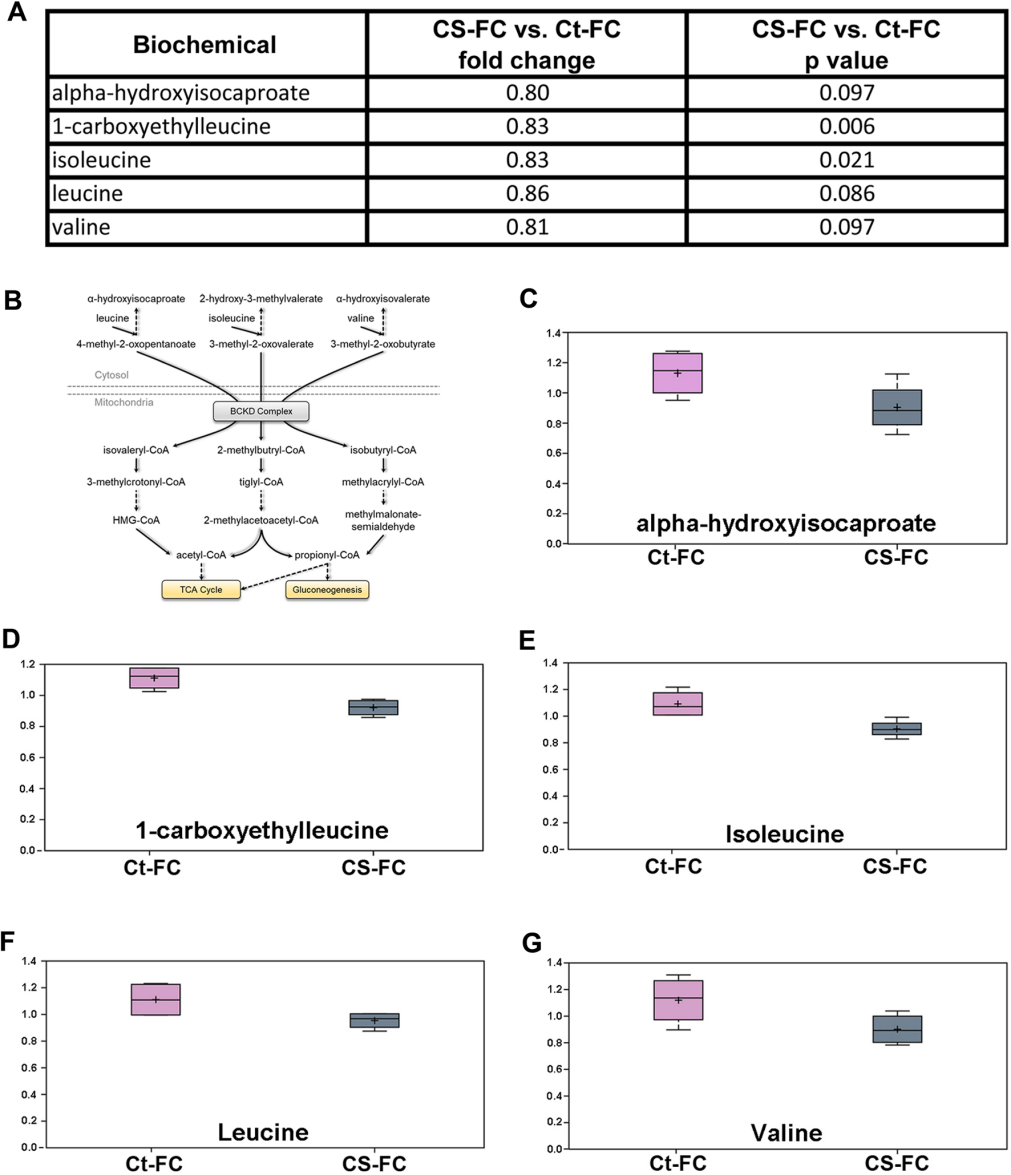
Metabolome analysis revealed diminished levels of branched-chain amino acids (BCAAs)-related metabolites in mouse lens fiber cells (FC) exposed to cigarette smoke (CS). (**A)** The diminished levels of BCAAs-related metabolites (alpha-hydroxyisocaproate, 1-carboxyethylleucine, isoleucine, leucine, and valine) identified in lens FC of mice exposed to CS. (**B)** Schematic of the pathway involving branched-chain α-keto acid dehydrogenase (BCKD), a multi-enzyme complex, which catalyzes BCAAs to the corresponding acyl-CoA derivatives. (**C–G)** Box plots depicting relative levels of BCAAs metabolites in lens FC of CS-exposed and untreated Ct mice. Diminished levels of **(C)** alpha-hydroxyisocaproate, **(D)** 1-carboxyethylleucine, **(E)** Isoleucine, **(F)** Leucine, and **(G)** Valine were identified in lens FC of mice exposed to CS.

Next, we completed the mass spectrometry-based metabolome profiling of lens FC of mice exposed CS. We identified a total of 280 metabolites in lens FC of mice exposed to CS (Supplementary Data 3). Among the 280 metabolites identified in lens FC of mice exposed to CS, we detected the differential levels of 14 metabolites (p < 0.05), including eight metabolites exhibiting higher levels and six metabolites displaying lower levels (Supplementary Data 3). We identified a decrease in branched-chain amino acid (BCAA)-related metabolites in lens FC of mice exposed to CS (Fig. 4A) including isoleucine, leucine, and valine, which are essential amino acids with key roles in protein synthesis, energy metabolism, and cell signaling^20^.

The BCAA metabolites have reported being involved in oxidative metabolism and energy production through a catabolic pathway involving branched-chain α-keto acid dehydrogenase (BCKD), a multi-enzyme complex, which catalyzes BCAA to the acyl-CoA derivatives (Fig. 4B)^21,22^. We identified diminished levels of alpha-hydroxyisocaproate, 1-carboxyethylleucine, isoleucine, leucine, and valine metabolites in lens FC of mice exposed to CS (Fig. 4C-G). In a recent study, Tomoda and colleagues analyzed the levels of BCAA-related metabolites and identified a significant decreased in BCAA levels in both plasma and skeletal muscles of rats exposed to CS^23^. We further detected a decrease of 1-carboxyethylleucine in lens FC of mice exposed to CS. Importantly, Das et al. reported the role of leucine and its transporters in protection against CS‐induced cell death in the lungs^21^.

Finally, we searched for the metabolome data to identify metabolites that are trending in the same direction i.e., ﻿elevated and/or diminished levels in lens FC of mice exposed to CS but are not statistically significant. The analysis revealed elevated levels of twenty-eight metabolites associated with long‐chain fatty acids, lysophospholipids, monoacylglycerols, and endocannabinoids in lens FC of mice exposed to CS (Supplementary Table 1). In parallel, decreased levels of 20 metabolites associated with phosphatidylethanolamine (PE), phosphatidylserine (PS), pentose metabolism, Fructose, Mannose, and Galactose Metabolism, and nucleotide Sugar in lens FC of mice exposed to CS (Supplementary Table 2). The low counts of sugar metabolism-related metabolites are inline with the low levels of BCAA related metabolites, involved in oxidative metabolism and energy production^22^.

Since we observed a minimal effect of CS exposure on the molecular landscape of lens FC with no cataracts in mice exposed to CS, we sought evidence of CS exposure by examining Cadmium (Cd), and Nickel (Ni) in the lungs of mice exposed to CS. The premise of the experiment was based on the results of two independent studies reporting a 3.1- and 1.7-fold increase in Cd concentration in the serum of tobacco cigarette smokers^24,25^. The premise was also based on the identification of Cd and Ni in mainstream particulates of cigarettes containing tobacco^26^.

We performed total metal quantification in lungs of mice exposed to CS using an Agilent 7500cx inductively coupled plasma mass spectrometer. The analysis identified a 3.70-fold (*p* = 0.0001) and a 1.54-fold (*p* = 0.01) higher concentration of Cd and Ni, respectively in the lungs of mice exposed to CS compared with untreated Ct mice (Table 1 and Supplementary Table 3). The statistically significantly higher concentrations of Cd and Ni in the lungs of mice exposed to CS are in line with chronic exposure to CS.

**Table 1:** Mass spectrometry-based metal ions analysis of lungs from mice exposed to cigarette smoke (CS) and age-matched, untreated control mice. The quantification shows differential concentrations of cadmium (Cd), and nickel (Ni) in the lungs of mice exposed to CS.

Sample	Cadmium		Nickel	
	Concentration (ppb)	RSD	Concentration (ppb)	RSD
Ct-ml-1	0.253	26.0	12.150	10.2
Ct-ml-2	0.519	19.0	16.455	4.8
Ct-ml-3	0.196	19.5	9.977	7.7
Ct-ml-4	0.538	12.1	19.550	8.3
CS-ml-1	1.487	3.6	20.152	5.1
CS-ml-2	1.366	8.6	20.863	2.0
CS-ml-3	1.541	10.1	23.134	8.7
CS-ml-4	1.178	8.2	25.652	5.0

The metal ion concentrations are normalized for sample mass and dilution. The metal ion concentrations are a mean value of five scans. RSD (relative standard deviation) is standard deviation divided by the mean value of the five scans (concentration in Table 1) and reported as a percentage. The detection limits were 0.2, and 2.0 ppb for Cd, and Ni respectively.

*Ct-ml* control mouse lungs, *CS-ml* cigarette smoke-exposed mouse lungs, *ppb* parts per billion (also equilment to ng/g).

In summary, we report results of a multi OMIC approach to examine the molecular landscape of mice lens FC exposed to CS for 3½ months, which confirmed a minimal effect on the molecular landscape of lens FC. However, we cannot rule out that CS exposure for longer durations (i.e., > 3½ months) and/or in combination with other morbidities or environmental factors would have a more robust effect and/or result in cataractogenesis.

## Materials and methods

### Animals included in the study

The use of mice in this study was approved by the Johns Hopkins Animal Care and Use Committee (ACUC; Baltimore, MD, USA), and all experiments were performed in accordance with the approved protocol by the Institutional Review Board (IRB) of the Johns Hopkins University School of Medicine (Baltimore, MD) and consistent with the Association of Research in Vision and Ophthalmology (ARVO) statement for the use of animals in ophthalmic and vision research. The C57BL/6J mice strain (Stock # 000664; Jackson laboratory) was used for all experiments.

The study design included placing pregnant mice (gestation days 18–20) in a whole-body smoke chamber and a few days later pups were born. Of these, four adult females along with 32 pups (16 males and 16 females) were exposed to CS for 5 hours/day, 5 days/week, and remained in the chamber for a total of 3½ months (105 days). In parallel, age-matched, four adult female mice and 32 (16 males and 16 females) pups were housed in the animal facility for 3½ months that served as untreated controls.

### Exposure to CS in a whole-body smoke chamber

The mice were exposed to CS as described^27,28^. The smoke chamber contained a smoking machine (TE-10, Teague Enterprises, Davis, CA) that burns five cigarettes (2R4F reference cigarettes (2.45 mg nicotine/cigarette; Tobacco Research Institute, University of Kentucky) at a time, taking two-second duration puffs at a flow rate of 1.05 l/min, to provide a standard puff of 35 cm^3^, providing a total of eight puffs per minute. The machine was adjusted to produce sidestream (89%) and mainstream smoke (11%). The chamber atmosphere was monitored to maintain total suspended particulate at 90 mg/m^3^ and carbon monoxide at 350 ppm.

### Evaluation of lens phenotype in CS-exposed and untreated Ct mice

The mice were examined for CS-related cataractogenesis after completion of CS exposure^29^. The eyes of CS-exposed and untreated CT mice were dilated using tropicamide (1%) and phenylephrine (2.5%) followed by anesthetization by ketamine/xylazine (100 mg/kg body weight for ketamine and 16 mg/kg body weight for xylazine). The lenses were examined with a slit-lamp microscope and images were taken by Phoenix Micron III Retinal Imaging Microscope (Phoenix Research Labs, Pleasanton, CA, USA).

### Extraction of the lenses and separation of lens FC

The mice were anesthetized with isoflurane and euthanized through cervical dislocation. The lenses were extracted from CS-exposed and untreated Ct mice and FC were separated from the lens epithelium using forceps under a microscope. The lens FC from CS-exposed and untreated Ct mice was maintained at − 80 °C in distinct pools (i.e., biological replicates), each consisting of FC of the right eye or the left eye.

We used four biological replicates (two male and two female) of the CS-exposed and untreated Ct mice, each consisting of lens FC of the right eyes for transcriptome profiling and their respective left eyes for proteome profiling. As a minimum of 25 mg of lens FC mass was required for metabolome analysis, each of the four biological replicates of the CS-exposed and untreated Ct mice consisted of eight lens FC (both right and left eyes) from four male or four female mice.

### Transcriptome profiling

Next generation RNA-Seq of lens FC of CS-exposed and untreated Ct mice was performed commercially by Novogene Corporation Inc. (Sacramento, CA). A total of four biological replicates, each consisting of pooled lens FC of the right eyes of four CS-exposed and untreated Ct mice was used for transcriptome profiling and the RNA-Seq data were analyzed as described^29^.

The FPKM expression values were imported in the Spotfire DecisionSite with Functional Genomics (TIBCO Spotfire, Boston, MA) software for further evaluation and graphical representation. All transcripts log2-fold changes between lens FC of CS-exposed and untreated Ct mice were analyzed to determine the SD from their mean of 0, which represents no change.

### Proteome profiling

Four biological replicates, each consisting of pooled lens FC of the left eyes of four CS-exposed mice and four untreated Ct mice were used for proteome profiling. A total of 200 μg of protein for each sample was used for each replicate. The reduction and alkylation of the proteins were conducted with 10 mM of tris (2-Carboxyethyl) phosphine hydrochloride and 40 mM of chloroacetamide for 1 h at room temperature. Mass spectrometry-based proteome profiling was completed as described^14^.

The abundance values of reporter ion intensities from the 8-plex TMT experiment were imported into Partek Genomics Suite v6.6 (Partek, Inc., St. Louis, MO, USA) for protein annotation and differential expression analysis. The normalized reporter ion intensities were examined for SD to investigate the differential expression in lens FC of mice exposed to CS compared with lens FC of untreated Ct mice. The p values were estimated by a two-tailed t-test, assuming a hypothesized mean of 0 change. The normalized ratios were converted to a log_2_ scale (becoming the conventional “log-ratios” or “log2 fold changes”) for statistical and graphic representation.

### Metabolome profiling

Four biological replicates, each consisting of pooled lens FC, wet mass (25 mg) of four CS-exposed and untreated Ct mice eyes (right and left) were used for metabolome profiling. The FC were frozen immediately at − 80 °C until further processing for metabolome profiling. Metabolome profiling and data analysis were performed commercially by Metabolon, Inc. (Morrisville, NC, USA) as described^30^.

Sample preparation involved metabolite extraction with methanol and the resulting extracts were analyzed on an accurate mass global metabolomics platform consisting of multiple arms differing by chromatography method and mass spectrometry ionization mode^30,31^. Metabolites were identified by automated comparison of the ion features in the experimental samples to a reference library of chemical standard using software developed at Metabolon^32,33^.

### Metal ion analysis

Metal ion quantification and data analysis were performed by the Biomarker Mass Spectrometry Facility (UNC-Chapel Hill, NC). A total of four biological replicates, each consisting of pooled mice lungs wet mass (250–460 mg) of four CS-exposed and untreated Ct mice were used for metal ion analysis. Mouse lungs were frozen immediately at − 80 °C upon extraction, until further processing. The samples were digested with a combination of concentrated nitric acid, 30% hydrogen peroxide, and heat. The digested samples were diluted to 4 ml with deionized water. Quantification of total Cd, and Ni, was performed using Agilent 7500cx inductively coupled plasma mass spectrometer (ICP-MS; Santa Clara, CA) as described^34^.

### ARRIVE guidelines

This study was completed in compliance with the ARRIVE guidelines.

## Supplementary Information

Supplementary Information 1.

Supplementary Information 2.

Supplementary Information 3.

Supplementary Tables.

## Data Availability

RNA-Seq raw reads and processed data of lens FC of CS-exposed and untreated Ct mice have been deposited in the NCBI Gene Expression Omnibus and are accessible through the GEO accession number GSE144818. The mass spectrometry data of lens FC of CS-exposed and untreated Ct mice have been deposited to the ProteomeXchange Consortium via the PRIDE partner repository with the dataset identifier PXD017414 and PXD020421. The metabolome raw data of lens FC of CS-exposed and untreated Ct mice have been deposited in the MetaboLights repository and are accessible through study identifier MTBLS207.
