# Supplementary material for: Examining the effects of cigarette smoke on mouse lens through a multi OMIC approach
Source: Sci Rep. 2021 Sep 22;11:18801. doi: 10.1038/s41598-021-95013-7 (PMC8458305; doi:10.1038/s41598-021-95013-7)
Supplement: Supplementary file 4 — Supplementary Tables. [file 41598_2021_95013_MOESM4_ESM.docx]

**Examining the effects of cigarette smoke on mouse lens through a multi OMIC approach**

Shahid Y. Khan ^1^, Muhammad Ali ^1^, Yura Jang ^2^, Taekyung Ryu ^2^, Andrew J. Schwab ^3^,

Brian O. Ingram ^3^, Peter H. Cable ^4^, Chan Hyun Na ^2^, James T. Handa ^1^ & S. Amer Riazuddin ^1^

^1^The Wilmer Eye Institute, Johns Hopkins University School of Medicine, Baltimore, MD 21287, USA. ^2^Department of Neurology, Institute for Cell Engineering, Johns Hopkins University School of Medicine, Baltimore, MD 21205, USA. ^3^Metabolon Inc., Morrisville, NC 27560, USA. ^4^Department of Environmental Sciences and Engineering, University of North Carolina, Chapel Hill, NC 27599, USA.

| **Sub-pathway** | **Biochemical** | **CS-FC vs. Ct-FC**  **p-value** | **CS-FC vs. Ct-FC**  **Fold-change** |
| --- | --- | --- | --- |
| Long Chain Monounsaturated Fatty Acid | oleate/vaccenate (18:1) | 0.4936 | 1.51 |
|  | eicosenoate (20:1) | 0.6149 | 1.36 |
|  | erucate (22:1n9) | 0.6041 | 1.39 |
| Long Chain Polyunsaturated Fatty Acid (n3 and n6) | docosapentaenoate (n3 DPA; 22:5n3) | 0.5765 | 1.72 |
|  | docosahexaenoate (DHA; 22:6n3) | 0.614 | 1.32 |
|  | dihomo-linoleate (20:2n6) | 0.3471 | 1.73 |
|  | arachidonate (20:4n6) | 0.5122 | 1.33 |
|  | docosapentaenoate (n6 DPA; 22:5n6) | 0.7623 | 1.91 |
|  | docosadienoate (22:2n6) | 0.6182 | 1.42 |
|  | mead acid (20:3n9) | 0.5206 | 1.71 |
| Lysophospholipid | 2-palmitoleoyl-GPC (16:1)* | 0.0286 | 1.48 |
|  | 1-palmitoyl-GPE (16:0) | 0.4556 | 1.64 |
|  | 1-stearoyl-GPE (18:0) | 0.7606 | 1.18 |
|  | 2-stearoyl-GPE (18:0)* | 0.3152 | 1.81 |
|  | 1-oleoyl-GPE (18:1) | 0.2451 | 1.22 |
|  | 1-palmitoyl-GPS (16:0)* | 0.2904 | 1.90 |
|  | 1-stearoyl-GPS (18:0)* | 0.5178 | 1.60 |
|  | 1-oleoyl-GPS (18:1) | 0.1966 | 1.73 |
|  | 1-oleoyl-GPG (18:1)* | 0.4190 | 1.50 |
|  | 1-oleoyl-GPI (18:1) | 0.4217 | 1.51 |
|  | 1-arachidonoyl-GPI (20:4)* | 0.0953 | 1.74 |
| Monoacylglycerol | 1-palmitoylglycerol (16:0) | 0.2505 | 1.43 |
|  | 1-oleoylglycerol (18:1) | 0.1323 | 1.74 |
|  | 1-arachidonylglycerol (20:4) | 0.0952 | 2.85 |
| Endocannabinoid | oleoyl ethanolamide | 0.4666 | 1.51 |
|  | N-oleoyltaurine | 0.5900 | 1.40 |
|  | N-stearoyltaurine | 0.6145 | 1.40 |
|  | N-palmitoyltaurine | 0.6376 | 1.34 |

**Supplementary Table 1:** Metabolites exhibiting elevated levels (non-significant) in cigarette smoke-exposed mice lens fiber cells compared to age-matched, untreated control mice lens fiber cells.

**Note:** Ct-FC: age-matched, untreated control mice lens fiber cells; CS-FC: cigarette smoke-exposed mice lens fiber cells.

| **Sub-pathway** | **Biochemical** | **CS-FC vs. Ct-FC**  **p-value** | **CS-FC vs. Ct-FC**  **Fold-change** |
| --- | --- | --- | --- |
| Phosphatidyl-ethanolamine (PE) | 1-palmitoyl-2-oleoyl-GPE (16:0/18:1) | 0.5958 | 0.95 |
|  | 1-palmitoyl-2-docosahexaenoyl-GPE (16:0/22:6)* | 0.1616 | 0.80 |
|  | 1-palmitoleoyl-2-oleoyl-GPE (16:1/18:1)* | 0.3967 | 0.92 |
|  | 1-stearoyl-2-oleoyl-GPE (18:0/18:1) | 0.5539 | 0.94 |
|  | 1-stearoyl-2-docosahexaenoyl-GPE (18:0/22:6)* | 0.1018 | 0.81 |
|  | 1,2-dioleoyl-GPE (18:1/18:1) | 0.5642 | 0.95 |
|  | 1-oleoyl-2-docosahexaenoyl-GPE (18:1/22:6)* | 0.2611 | 0.85 |
| Phosphatidylserine (PS) | 1-palmitoyl-2-oleoyl-GPS (16:0/18:1) | 0.2805 | 0.68 |
|  | 1-palmitoyl-2-linoleoyl-GPS (16:0/18:2) | 0.2244 | 0.55 |
|  | 1-stearoyl-2-oleoyl-GPS (18:0/18:1) | 0.2025 | 0.58 |
|  | 1,2-dioleoyl-GPS (18:1/18:1) | 0.3439 | 0.76 |
| Pentose Metabolism | ribonate | 0.2745 | 0.9 |
|  | arabonate/xylonate | 0.9426 | 0.99 |
|  | sedoheptulose | 0.105 | 0.84 |
|  | ribulonate/xylulonate/lyxonate* | 0.8186 | 0.91 |
| Fructose, Mannose, and Galactose Metabolism | mannitol/sorbitol | 0.3606 | 0.93 |
| Nucleotide Sugar | UDP-glucose | 0.3678 | 0.92 |
|  | UDP-galactose | 0.4327 | 0.93 |
|  | UDP-glucuronate | 0.6588 | 0.89 |
|  | guanosine 5'-diphospho-fucose | 0.8207 | 0.95 |

**Supplementary Table 2:** Metabolites exhibiting diminished levels (non-significant) in cigarette smoke-exposed mice lens fiber cells compared to age-matched, untreated control mice lens fiber cells.

**Note:** Ct-FC: age-matched, untreated control mice lens fiber cells; CS-FC: cigarette smoke-exposed mice lens fiber cells.

**Supplementary Table 3:** Metal ions quantification shows statistically significant fold-change of Cadmium, and Nickel ion concentrations in the lungs of CS-exposed mice compared to the lungs of age-matched, untreated control mice.

| **Metal Ions** | **CS-ml vs. Ct-ml (fold-change)** | **CS-ml vs. Ct-ml (p-value)** |
| --- | --- | --- |
| Cadmium (Cd) | 3.70 | 0.0001 |
| Nickel (Ni) | 1.54 | 0.01 |

**Note:** We used a two-tailed student`s *t*-test for statistical analysis. Ct-ml: age-matched, untreated control mice lungs; CS-ml: cigarette smoke-exposed mice lungs.

**Supplementary Data 1:** The list of genes identified in cigarette smoke (CS)-exposed and age-matched, untreated control (Ct) mice lens fiber cells (FC).

**Supplementary Data 2:** The list of proteins identified in cigarette smoke (CS)-exposed and age-matched, untreated control (Ct) mice lens fiber cells (FC).

**Supplementary Data 3:** The list of metabolites identified in cigarette smoke (CS)-exposed and age-matched, untreated control (Ct) mice lens fiber cells (FC).
